# Brain Ischemia in Alzheimer’s Disease May Partly Counteract the Disruption of the Blood–Brain Barrier

**DOI:** 10.3390/brainsci15030269

**Published:** 2025-03-02

**Authors:** Grant A. Bateman, Alexander R. Bateman

**Affiliations:** 1 Department of Medical Imaging, John Hunter Hospital, Newcastle, NSW 2310, Australia; 2 School of Medicine and Public Health, College of Health, Medicine and Wellbeing, Newcastle University, Callaghan Campus, Newcastle, NSW 2308, Australia; 3 School of Engineering, College of Engineering, Science and Environment, Newcastle University, Callaghan Campus, Newcastle, NSW 2308, Australia; alex.bateman@newcastle.edu.au

**Keywords:** Alzheimer’s disease, blood–brain barrier, cerebral blood flow, CSF formation rate, glymphatic, ischemia, normal pressure hydrocephalus

## Abstract

Background: In normal pressure hydrocephalus (NPH) there is blood–brain barrier (BBB) disruption, which should increase the CSF formation rate (CSF_fr)_ and, therefore, also increase the intracranial pressure (ICP). However, the ICP is normal in NPH. A lumped parameter study was performed to look at the interrelation between the ICP, cerebral blood flow (CBF), and the degree of BBB disruption in NPH. The model suggested that the CSF_fr_ could be reduced in this condition if the BBB disruption was moderated by a reduction in the capillary transmural pressure (TMP) secondary to arteriolar constriction and a reduced CBF. In early Alzheimer’s disease (AD), there is BBB disruption, reduced ICP, and global ischemia. This raises the possibility that the same physiology may occur in AD as occurs in NPH. Methods: A lumped parameter model previously used to describe the hydrodynamics of NPH was modified to investigate the effects of changes in CSF pressure and blood flow in patients with mild cognitive impairment (MCI) and AD. Results: The model indicates that the average capillary TMP is normal in MCI, but decreases as AD progresses. Removing CSF in AD patients during a tap test initially increases the capillary TMP. The brain in AD responds to a tap test by increasing its level of ischemia, and this reduces the capillary TMP. Conclusions: A hypothesis is put forward that the BBB disruption in AD is partially mitigated by the brain making itself ischemic. Modelling gives support to this hypothesis. The model can suggest a cause for the development of ischemic neuronal loss and amyloid accumulation secondary to glymphatic flow disruption as AD progresses.

## 1. Introduction

Alzheimer’s disease (AD) is the most common cause of dementia in the elderly population. It is characterised by brain atrophy and gradual cognitive decline, which correlates with a loss of neuronal synapses and cell death [1]. The exact pathophysiology of this disease remains difficult to discern. The blood–brain barrier is a concept whereby the capillary walls of the brain are known to be very impermeable to the passage of water, salt, and proteins compared to the rest of the systemic circulation. There is evidence to suggest that BBB disruption occurs early in AD. In a study of mild cognitive impairment (MCI) due to AD and early AD using an MRI gadolinium contrast protocol, there was global leakage of contrast, with a higher volume fraction of leaking brain tissue in the cortex, deep grey matter, and normal-appearing white matter correlating with disease stage [2]. In both MCI and early AD, the mini mental state examination (MMSE) decreases significantly with the increasing leakage of the BBB in the grey matter [2]. The BBB protects the brain from blood-derived toxic molecules, cells, and microorganisms, and also regulates the transport of nutrients and the clearance of metabolic end products and endogenous neurotoxins [3]. The fact that the BBB breakdown occurs early in individuals with MCI and AD [4], even preceding hippocampal degeneration [5], suggests that it may be an initiating event. In acute brain insults such as cerebritis, there is the production of vasogenic oedema [6]. Vasogenic oedema is due to BBB disruption and results in the extravasation of fluid and intravascular proteins such as albumin into the cerebral parenchyma [6]. The intracranial pressure (ICP) is a measure of the pressure within the cranium compared to the air pressure. The excessive accumulation of oedema fluid evokes an increase in the ICP because there is continuity between the interstitial and the subarachnoid spaces [6]. Therefore, BBB breakdown in AD would be expected to increase the ICP. However, the opposite occurs, with a reduction in the ICP in AD over time. In a large study measuring the ICP in MCI and AD, there was a significant linear correlation between the reduction in the MMSE and a reduced ICP across the whole cohort [7]. Thus, the progressive reduction in the ICP, despite the progressive opening of the BBB in AD, appears to be an enigma.

There is a high correlation between the pathology of AD and normal pressure hydrocephalus (NPH) found at brain biopsy performed whilst inserting a shunt to treat NPH [8]. The syndrome of NPH was first described by Adams et al. almost 60 years ago, in patients with a classical clinical triad of ataxia, incontinence, and dementia [9]. These patients were found to have dilated ventricles but normal cerebrospinal fluid (CSF) pressure [9]. The ICP is normal in NPH despite there being a significant disruption of the BBB [10]. As discussed, this suggests an anomaly in NPH pathophysiology similar to AD. Thus, there may be some pathophysiological overlap between NPH and AD. A lumped parameter hydrodynamic study of the brain in NPH performed by the current authors was used to investigate this anomaly. This study suggested that the expected increased ICP in NPH could be moderated if the capillary pressure was reduced by arterial constriction, leading to cerebral ischemia [11]. Interestingly, reduced cerebral blood flow (CBF) is also found to be a component of AD. Cerebral blood flow is a measure of the amount of blood entering the brain per minute per 100 g of tissue. A study by one of the current authors showed the mean arterial pressure to be the same in a cohort of AD patients as compared to aged-matched controls. Using 2D phase contrast MR flow quantification, the total arterial inflow in the AD patients was measured to be 18% lower than the controls. Dividing the total blood flow by the mean arterial pressure suggested that the vascular resistance in AD was increased by 23% (*p* = 0.02) compared to the controls [12]. This indicated a probable significant arterial dysfunction in AD, provided the capillary or venous pressures were not affected. In a large study, decreased global CBF was associated with worse cognitive performance in AD and impairment in all cognitive domains [13]. There is a linear reduction in both CBF and the MMSE in AD patients over time, with a strong relationship between the decrease in global CBF and cognition [14]. Thus, there are similarities between the breakdown in the BBB, reduced or moderated ICP, and evidence of cerebral ischemia between NPH and AD. This suggests to us a possible hypothesis, i.e., the brain is partly inducing ischemia within its parenchyma in AD as a way of moderating the effects of the BBB disruption in a way similar to NPH. This hypothesis could be investigated further using the lumped parameter model we have previously developed. Therefore, the purpose of the current study is to extend the original lumped parameter model in order to incorporate the ICP and ischemia in AD to test the hypothesis that the brain is reducing its blood flow as a way of moderating the ICP and the effects of the disruption of the BBB. If a mechanism establishing the interaction between the alterations found in the ICP, BBB, and CBF in Alzheimer’s disease could be established, this may allow both prognostic information and the ability to measure the effect of therapeutic interventions using one of the variables alone (such as the CBF) as a surrogate marker of the other two variables.

## 2. Materials and Methods

A detailed description of the model can be obtained from the original and follow-up papers [11,15]. A brief description is given to outline the methods used.

### 2.1. Equations

Davson’s equation relates the intracranial pressure to the CSF formation rate, the CSF outflow resistance, and the venous sinus pressure [16]:

(1) $$ICP={CSF}_{fr}\times R_{out}+P_{sss}$$

where ICP is the intracranial pressure, CSF_fr_ is the CSF formation rate, R_out_ is the CSF outflow resistance, and P_sss_ is the pressure in the superior sagittal sinus. Next, Ohms law for hydraulic circuits is required:

(2) ∆P=Q×R

where ΔP is the pressure drop across a vascular segment, Q is the flow rate through the segment, and R is the resistance. Resistances in series are additive, so the following can be derived:

(3) $$R_{art}+R_{cap}+R_{ven}+R_{cuf}=R_{tot}$$

where R_art_ is the arterial segment resistance, R_cap_ is the resistance in the capillaries, R_ven_ is the venous resistance, R_cuf_ is the resistance of the venous outflow cuff, and R_tot_ is the total resistance for the entire vascular system. Poiseuille’s equation calculates the pressure drop across each of these segments:

(4) ΔP=8μLQπr4

where ΔP is the pressure drop, µ is the viscosity, L is the vessel length, Q is the fluid flow rate, π is the circle proportionality constant, and r is the radius. Substituting Equation (2) into (4) and eliminating Q from both sides gives the following equation for the resistance in each segment.

(5) R=8μLπr4

As the volume of a tube is proportional to the radius squared, the following can be shown.

(6) $$∆R=∆V^{-2}$$

It has been previously shown that the volume of the venous outflow varies with the transmural pressure using the following equation [11]:

(7) $${∆TMP}_{ven}=-0.033{{\Delta V}_{ven}}^{2}+7.49\times {\Delta V}_{ven}-3.44$$

where ΔTMP_ven_ is the normalised increase in venous transmural pressure and ΔV_ven_ is the change in venous volume. The pressure within the venous outflow sinuses depends on the central venous pressure and the pressure drop across the venous sinuses to the level of the jugular bulbs. The normal central venous pressure is 5 ± 0.7 mmHg [17]. Although the pressure drop across the venous outflow has been found to have a quadratic relationship with the CBF [18], the relationship of the portion of the graph between a normal flow rate and zero flow is almost linear. Thus, the venous sinus pressure can be found using the following simplified equation:

(8) $$P_{sss}=3.3\times CBF+5$$

where P_sss_ is the pressure in mmHg and CBF is the blood flow in L/min.

### 2.2. Model Input Parameters

The input parameters are unchanged from the previous studies [11,15] and will only be briefly described, as the details can be obtained from the original study. The brain size is 1500 g. The cerebral blood arterial inflow is 750 mL/min. The arterial inflow pressure is 100 mmHg [19]. The precapillary bed pressure is 32 mmHg [20]. The end capillary pressure is 15 mmHg [21]. The CSF pressure is 11.5 mmHg [22] and the pressure gradient from the CSF to the superior sinus lumen is 4 mmHg [23], giving a sinus pressure of 7.5 mmHg [24]. The transmural pressure of the subarachnoid cortical veins in primates is 2.5 mmHg [25]. The pre-venous outflow cuff pressure is 14 mmHg by addition.

The total cerebral blood volume (CBV) is 51 mL [11]. The arterial component of the CBV is 25% of the total [26] or 12.8 mL. The capillaries make up 53% of the remaining volume [27], giving a total capillary blood volume of 20.3 mL and a total venous blood volume of 17.9 mL. The normal CSF formation rate is 0.40 mL /min [28].

### 2.3. Vessel Responses to Transmural Pressure Variations

The variations in the arterial resistance and volume in this model depend entirely on the arterial muscle tone and not the vessel transmural pressure. As the arterial pressure is always much higher than the ICP, the arterial transmural pressure will have no effect on the outcome of the current modelling study.

The capillary bed vessels do not actively alter their diameter [29], indicating that they react purely to their transmural pressure. To simplify the current study, it is assumed that the volume of the capillaries varies between normal and their maximally dilated volume as a linear function of their transmural pressure. A previous study indicated that an increase in capillary TMP from 12 to 37.9 mmHg would increase the capillary volume by 44% or a 1.7% increase in volume for each 1 mmHg pressure rise [11]. Below a TMP of 12 mmHg, the volume is unchanged at 20.3 mL, and above a TMP of 37.9, the elastic limit is reached and the volume is set to 29.2 mL. The lack of a reduction in capillary volume below a normal TMP of 12 mmHg is due to the very high bending forces that would be required to buckle such small vessels [30].

Similar to the capillaries, the veins alter their size purely depending on their transmural pressures. In a previous modelling study [11], the function for the outflow vein dilatation was found to be summarised by Equation (7).

At the distal end of the cortical veins, as they join the sinus wall, the outflow cuff segment resides. The collapse of this segment occurs physiologically and is passively modulated by the transmural pressure between the ICP and the sinus pressure, which is usually negative [31]. The segment is very short, and as it is mostly under a state of collapse with physiological ICPs; the change in volume from this segment will be ignored in this model. This is despite acknowledging that the cuff is dilated in the tap test model. However, its resistance will be taken into consideration. In the previous study, four differing cuff transmural pressures resulted in four differing resistances [11]. When these points were plotted, a straight line with an R^2^ of 0.998 resulted, suggesting the cuff resistance varies as a linear function of the cuff TMP, thus giving Equation (9):

(9) $$R_{cuf}=-2.71\times {TMP}_{cuf}+0.008$$

where R_cuf_ is the cuff resistance and TMP_cuf_ is the cuff transmural pressure.

The sagittal sinus pressure will vary with the blood flow using Equation (8).

## 3. Results

### 3.1. Varying Blood Flow and ICP in AD

**Figure 1:**
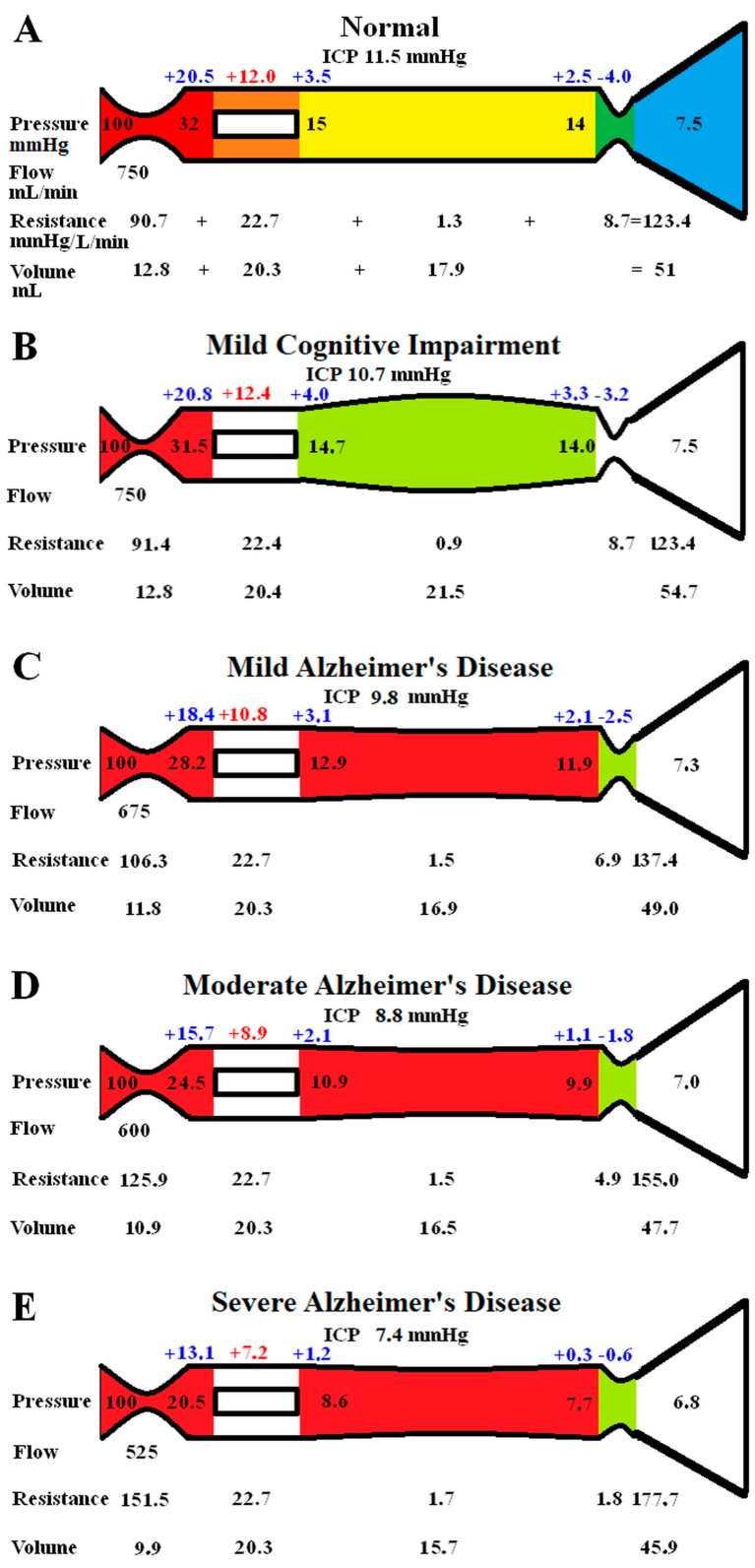
Results of modelling changes to blood flow in AD. (**A**) The normal findings. The segments from right to left are the arterial in red, the capillary in orange, the veins in yellow, the outflow cuff in green, and the venous sinus in blue. The vascular pressures are shown within the vessels. The numbers above the vessel are the transmural pressures at each site. The resistances and volumes for each segment are shown below the vessel. Note: ICP, intracranial pressure; mm, millimetres; mmHg, millimetres of mercury; mL, millilitres. (**B**) The findings in MCI. The red area indicates an increase in resistance in the arteries, and the green, decreased resistance in the veins compared to normal. (**C**) The findings in mild AD. The red area shows a further increase in arterial resistance and some increase in venous resistance, with the green area showing a reduction in cuff resistance. (**D**) The findings in moderate AD. These changes are essentially more pronounced than in mild AD. (**E**) The findings in severe AD. These changes have further progressed compared to earlier. Figure 1A has been reproduced from [11] under a CC BY 4.0 commons licence.

The original study findings in NPH suggested the brain elected to be ischemic, most likely to limit the capillary TMP and the CSF formation rate (CSF_fr_) [11]. We undertook an initial modelling study to see how the changes in CBF and ICP, known to occur in MCI and AD, would alter the capillary TMP to further study this effect. These initial modelling findings are summarised in Figure 1. The five vascular segments modelled are shown in Figure 1A; each segment has a differing colour. The segments from right to left are the arterial, the capillaries, the veins, the outflow cuff, and the sinus. The pressures obtained from the literature have been appended to the beginning and end of each vascular segment within the vessels in Figure 1A. Given the arterial inflow volume passes through each segment sequentially, the resistance of each segment can be calculated using Equation (2). These resistances are appended below the vessels in Figure 1. The normal cerebral blood volume (CBV) values for each segment and the total CBV has been obtained from the literature and are shown below the resistances. The numbers above the vessel represent the transmural pressure gradients between the pressure at the beginning and end of each capacitance vessel segment and the ICP, and are obtained by subtraction. The extra figure above the capillary (in red) is the mean TMP obtained by averaging the TMP before and after the capillaries. Placing the ICP, venous pressure, and normal CSF_fr_ in Equation (1) gives a normal R_out_ of 10 mmHg/mL/min. Figure 1B–E represent the effects of the differing alterations in CBF by varying the inflow resistance and ICP. In these figures, the red segments represent the areas of increased resistance compared to the normal findings, and the green represent reduced resistance.

In Figure 1B, the findings in MCI have been modelled. The arterial inflow is unchanged, as per the literature. The ICP is reduced to 10.7 mmHg, as per the literature. These pressure changes reduced the gradient pressure across the venous cuff, and the resistance of this segment was reduced very slightly. The reduction in ICP dilated the veins due to the change in their TMP. There were no other significant changes. Placing the ICP, venous pressure, and a normal CSF outflow resistance of 10 mmHg/mL/min into Equation (1) suggests the CSF_fr_ is reduced in MCI to 0.32 mL/min, down from 0.4 mL/min.

Figure 1C models the findings in mild AD. The ICP and the CBF come from the literature. The venous sinus pressure is reduced, the cuff pressure gradient is reduced, and the volume of the veins reduced. The effect was to reduce the capillary TMP below that for MCI. Placing the ICP, venous pressure, and the normal R_out_ into Equation (1) gave a CSF_fr_ of 0.25 mL/min, which was a further reduction compared to MCI.

Figure 1D models moderate AD with further reductions in ICP and CBF. The effect is similar to, but more pronounced than, the changes in mild AD, with the capillary TMP being further reduced. The calculated CSF_fr_ is also further reduced to 0.18 mL/min

Figure 1E models severe AD with further reductions in CBF and ICP. The capillary TMP is further reduced to 7.2 mmHg, and the calculated CSF_fr_ is 0.06 mL/min. Note that the total CBV has decreased by 10% compared to the normal figure.

### 3.2. Performing a Tap Test in Moderate AD

**Figure 2:**
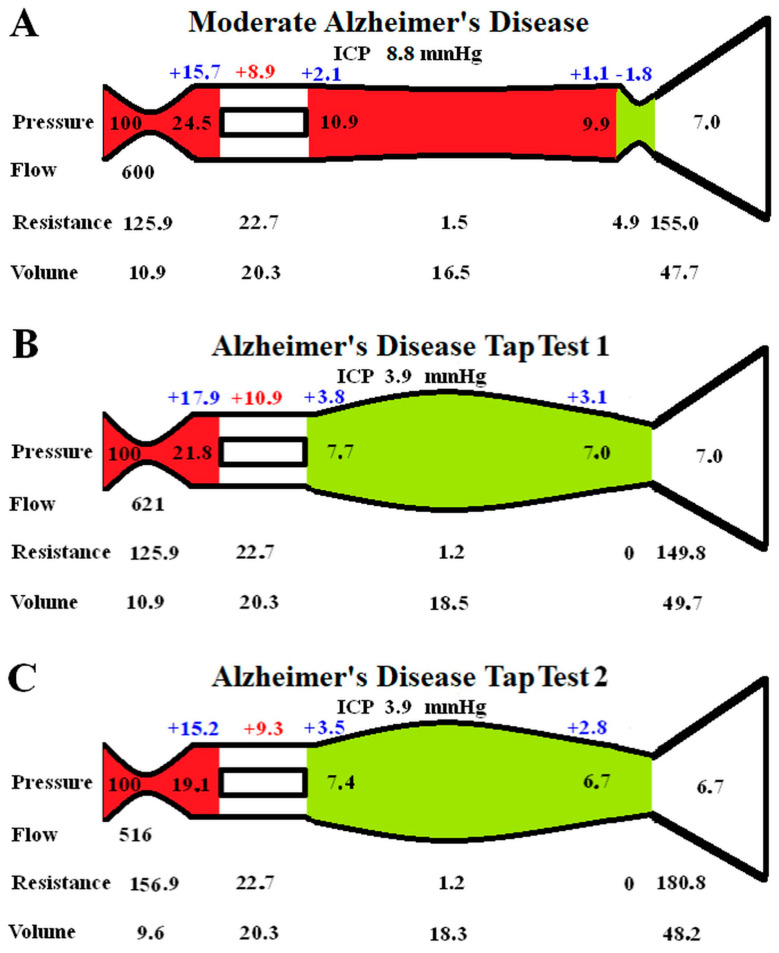
Modelling of changes secondary to a tap test in AD: (**A**) shows the moderate AD findings reproduced from Figure 1D for ease of comparison; (**B**) shows the immediate finding following a tap test. Note the veins dilate, reducing the venous pressure, and the flow increases slightly, but the capillary TMP has increased; (**C**) shows the findings in a tap test 1 min later as the CBF drops. This required an increase in the arterial resistance, reducing the size of the arteries. The veins are dilated by almost the same amount, meaning the total CBV is almost unaltered from Figure 2A.

The effect of performing a tap test in moderate AD is modelled in Figure 2. In Figure 2A, the baseline moderate AD findings are transposed from Figure 1D for easier comparison.

Figure 2B is the instantaneous effect of reducing the ICP by 56%, as per the literature. The outflow cuff resistance is abolished due to the now positive pressure gradient across it. The unchanged arterial resistance allows for a minimal increase in CBF of 3.5%. There is a reduction in venous pressure, but the ICP has dropped faster than this, so the capillary TMP increases by 22.5%. The green segment indicates that dilatation of the veins and reduced resistance.

According to the literature, the effect of a tap test is to induce a 10% reduction in CBF within one minute. This effect has been modelled in Figure 2C. The venous resistances are unchanged as they only depend on the transmural pressures, which are minimally changed. A significant increase in arterial resistance is required, this reduced the capillary TMP by 14.7% compared to Figure 1B because the precapillary pressure dropped and the venous sinus pressure dropped. The capillary TMP is reduced back towards the findings in Figure 2A. Note that the CBV is not significantly different from Figure 2A because the venous dilatation is matched by the arterial constriction.

## 4. Discussion

We hypothesised that the brain makes itself ischemic, in part, to reduce the effect of the BBB breakdown in AD and to moderate the ICP. We wished to test this using a previously developed lumped parameter model. This model was originally verified by predicting the cerebral blood volume (CBV) changes that would occur at the limits of autoregulation, utilising both an increase and decrease in perfusion pressure [11]. The model accurately predicted the CBV changes found in the literature for both human and animal experiments [11]. Similarly, we can check the accuracy of the current modelling in AD by comparing the predicted CBV with the literature. The model predicts a 10% reduction in CBV in severe AD. In a study of 16 patients with an MMSE averaging 16/30, the CBV was reduced by 11% in the cortex [32], suggesting that the model is accurate enough for the current purposes. The overall findings of the model add some weight to the hypothesis that the brain is making itself ischemic to limit the effect of BBB disruption and, therefore, the rise in the ICP. Firstly, the capillary transmural pressure is reduced sequentially as the degree of BBB disruption increases with AD progression. The reduction in capillary transmural pressure would be expected to moderate the ICP rise. Secondly, the expected increase in CBF, which should occur after improving the cerebral perfusion pressure by a tap test, would increase the capillary transmural pressure. The paradoxical reduction in blood flow, which actually occurs following the tap, reduces the capillary transmural pressure back towards normal, minimising the fluid leakage and the ICP. Thes findings will be further discussed below.

### 4.1. Cerebral Blood Flow in MCI and AD

A meta-analysis suggested no obvious changes in global CBF in MCI patients compared to controls [33]. Therefore, we did not change the CBF in our model for MCI. At first glance, a normal global CBF in MCI would appear to make our suggestion, that a reduced CBF is the earliest response to an opening of the BBB, unlikely. However, the perfusion changes in MCI are varied. In a recent MRI study, there was hypo-perfusion in the right middle frontal gyrus, right and left temporal gyrus, and right middle temporal gyrus, but hyper-perfusion in the right superior medial gyrus, left and right praecuneus, left superior parietal lobule, right superior frontal gyrus, and right cerebellum [34]. Thus, the high-flow areas may mask the effect of the low-flow areas in MCI at a global level. As previously discussed, there is a linear reduction in CBF over time in AD, with a close association between this reduction and the MMSE [14], suggesting that the CBF reduction is closely related to the stage of the disease. In studies of mild AD with an MMSE of 26/30, the global CBF is reduced by between 6 and 10% [35,36]. We chose the figure of a 10% reduction in CBF for mild AD in our model. Studies of the CBF in moderate AD suggest a reduction of between 18 and 21% [12,37,38]. Therefore, we chose a 20% reduction in CBF in moderate AD. There are few studies looking at severe AD and CBF. Tohgi et al. found a 25.7% reduction in the cortex and 17.8% in the white matter in severe AD [32]. However, the global CBF can be reduced by up to 42% in this disease [39]. Given the linear response of the MMSE to the CBF, we elected to use a reduction of 30% in our model for severe AD. These reductions in CBF appear to occur despite there being no significant change in the arterial blood pressure [12,38]. Therefore, we kept the inflow pressure normal in the model.

Theoretically, a progressive reduction in CBF in AD could be predominantly secondary to atrophy or reduced metabolic activity; however, published metabolic imaging suggests otherwise. Using Positron Emission Tomography and 15O labelled compounds, it is possible to measure the CBF in mL/100 g/min, cerebral metabolic rate of oxygen (CMRO2) per 100 g of tissue, oxygen extraction fraction (OEF), and CBV in mL/100 g [40]. CMRO2 measures the rate of aerobic glycolysis per 100 g of tissue and the OEF is the fractional extraction of oxygen transferred from the capillary to the nerve cells [40]. The cerebral metabolic rate of glucose (CMRglu) can be measured with a radio labelled glucose analogue that measures anaerobic glycolysis per 100 g of tissue [40].

When there is a fall in cerebral perfusion pressure, the first compensation measure the brain performs is to dilate the arterioles to decrease their resistance and maintain the CBF and CMRO2; this is stage 1 [40]. Thus, the CBV should increase in stage 1. We note that the CBV actually progressively decreases with disease progression in AD [32]. A progressive reduction in CBV means the arteries cannot be dilating in response to progressive ischemia, and this is an anomaly. When the arterial vasodilatation in ischemia is exhausted, the CBF will fall, but the CMRO2 will initially be maintained by increasing the oxygen extraction with an increase in OEF; this is stage 2. If perfusion pressure falls further, then infarction becomes a possibility [40]. In 10 patients with AD judged to be moderate in severity, there were reductions in CBF, CMRO2, and CBV, with an increase in OEF compared to controls [40]. There was a profound reduction in CMRglu, indicating reduced anaerobic glycolysis [40]. The findings were described as misery perfusion. Note that this misery perfusion occurs whilst the arteries apparently do not dilate because the CBV is low. In this study, the cerebrovascular reserve was assessed using 7% CO2 inhalation and hyperventilation to increase and decrease the CBF, respectively [40]. There was no difference in values between the AD patients and controls, indicating a preserved cerebrovascular reserve [40]. Another study found similar findings with large reductions in CBV and CBF, increased OEF, and reduced CMRO2 [32], and a third also indicated a 9% reduction in whole-brain CMRglu in early AD [41]. This highlights a large discrepancy in AD. There is misery perfusion with metabolic derangement which is not entirely due to atrophy (the reduction occurs per 100 g of tissue), but the arterioles are not fully dilated, as evidenced by the reduced CBV, and there is a preserved cerebrovascular reserve. Therefore, the inference is that arterial constriction has caused at least some of the ischemia in AD and the arteries have elected not to dilate, even though they have the capacity to do so. In fact, the modelling in Figure 2 suggests that the arterioles actively react with a further constriction if the capillary TMP increases, suggesting that reducing the TMP may be a greater imperative than rectifying the metabolic insult.

A tap test is performed in NPH patients by removing 20–30 mL of CSF at lumbar puncture. It has long been known that a tap test increases the CBF in NPH in those patients who improve clinically, but the CBF paradoxically falls in those who do not improve clinically [42]. In properly auto-regulated patients, increasing the cerebral perfusion pressure should not have an effect on the CBF. Even in idiopathic intracranial hypertension patients and those with a recent stroke, the CBF does not change with a tap test [43,44]. However, in one study where a tap test was performed on AD patients, the ICP was reduced by 56%, but the CBF fell by 14% within one minute. The CBV was not significantly changed [43]. In another study, the tap test reduced the CBF by a further 10% on top of the 14% reduction in the baseline CBF in AD patients compared to controls [45]. A fall in the CBF despite an improvement in the perfusion pressure is an obvious anomaly. To study this effect, we reduced the ICP by 56% in Figure 2B from the moderate AD baseline in Figure 2A. The immediate effect would be a reduction in the venous outflow resistance and a moderate increase in CBF due to the improved perfusion pressure, Figure 2B. However, the capillary TMP was increased by 22.5% in Figure 2B. Following one minute, the CBF was reduced, so we reduced the CBF by 10% of the normal flow rate in Figure 2C to model this. This brought the capillary TMP down to be much closer to the original figure in Figure 2A. Note that the final CBV in Figure 2C is only 1% larger than the baseline moderate AD value in Figure 2A, despite the venous volume increasing by 11%. This is because the arterial volume decreased by 12%, and this matched the venous increase. This CBV model prediction mirrors the findings of Meyer et al., in which the CBF fell but the CBV was unchanged in AD following a tap test [44]. Again, this suggests that our modelling is probably accurate enough for our current purposes. The interpretation is that the reduction in capillary TMP may be more important to the brain than the reduction in CBF that this requires. Interestingly, calcium channel blockers such as Nimodipine (which are known to increase the CBF) have been shown to have a partial therapeutic effect in AD [46]. However, the increased blood flow may come at the expense of a worsening BBB disruption.

### 4.2. The Blood–Brain Barrier, CSF Formation Rate, and the ICP

As already discussed, the ICP is reduced with disease progression in AD [7]. In the study by Yang et al., the mean ICP in MCI was 10.7 mmHg, the patients with severe AD had an ICP of 7.4 mmHg, and the average across all dementia patients (representing the level for moderate dementia) was 8.8 mmHg [7]. We used these figures for our study. Given the linear relationship of the ICP with dementia grade, the value for mild dementia we used was taken to be half way between MCI and a moderate dementia figure, i.e., 9.8 mmHg [7].

The intact BBB effectively excludes significant changes in net fluid production within the brain parenchyma. The normal CSF_fr_ is 0.4 mL/min [28]. Of this, 0.28 mL/min comes from the choroid plexus, 0.072 mL/min from the brain parenchyma, and 0.048 mL/min from glucose metabolism [47]. Therefore, the brain parenchyma produces some interstitial fluid, which becomes incorporated into the CSF production, at a rate of 0.0048 mL/100 g/min. This compares to the remainder of the body, where interstitial fluid production contributes to the lymph fluid. For the skin, the production rate is 0.24 mL/100 g/min, for muscle, it is 0.021 mL/100 g/min [48], and for the liver, it is approximately 0.05 mL/100 g/min [49], i.e., 2–3 orders of magnitude greater than the fluid production within the brain. The fluid production can significantly increase in the brain parenchyma if there is an opening of the BBB. Increased arterial pressure above the autoregulation cut-off gives cerebral hyper-perfusion, increased pressure within the capillaries and venules, the disruption of the BBB, oedema, and a raised ICP [29]. How much can a chronic disruption of the BBB increase the interstitial fluid production? In NPH, there is a BBB breakdown [10,50]. There is also a 14% reduction in the CBF in the cortex and a 40% reduction in the white matter in NPH [15]. The capillary TMP in the cortex was modelled to be 9% above normal; but, in the white matter, it was 33% below normal [15]. It was argued that an open BBB would make the cortex over-produce CSF, but the white matter would absorb CSF and the balance would result in a normal ICP [15]. Therefore, if this is true, we would expect there to be reversed flow in the aqueduct to accomplish this. Linstrom et al. measured the CSF flow through the aqueduct in normal controls using phase contrast MRI and found it to be antegrade (coming out of the ventricles) at 0.18 mL/min, but the flow was retrograde (going in) in 65% of patients with NPH who improved following shunt [51]. These patients had 1.49 mL/min entering their ventricles [51]. Given that grey matter makes up 65% of the brain, this would give a parenchymal interstitial fluid production rate for grey matter of 0.15 m/100 g/min, which is between the interstitial fluid production rate for the skin and liver, as discussed previously, and much larger than normal. If the brain were not absorbing this fluid into the deep white matter at the same rate, then the 1.49 mL/min would be added to the 0.33 produced by the choroid plexus and glucose metabolism. Using Davson’s equation (1), the resultant CSF_fr_ of 1.82 mL/min, together with a normal R_out_ of 10 mmHg/ mL/min and a normal sinus pressure of 7.5 mmHg, would give a resultant ICP of 25.7 mmHg in NPH, which is way above the normal range. Therefore, an open BBB such as in NPH should significantly increase the ICP, and not leave it in the normal range, as recorded. Therefore, something is counteracting this effect, and a similar finding may occur in AD. Note that we used a normal R_out_ of 10 mmHg/ mL/min in the moderate AD model and we used Davson’s equation to calculate a net CSF_fr_ of 0.18 mL/min. This compares to Silverberg et al.’s finding of a net CSF_fr_ of 0.2 mL/min in moderate AD [52], suggesting that our use of a normal R_out_ in AD is probably correct. The brain in NPH appears to make its deep white matter ischemic to allow the ICP to return to the normal range. In AD, unlike NPH, the maximal reduction in the capillary TMP is in the cortex and not the white matter [32], and so a predicted excess in CSF production would be moderated at its source. There is a progressive reduction in the ICP in MCI and with AD grade, as shown in Figure 1, and the net CSF_fr_ we estimated from Davson’s equation also reduces with MCI and with AD grade. This suggests that the progressive reduction in the capillary TMP we estimated could be both correcting the over-production of CSF and also reducing the net CSF production to a degree as well. Alternatively, the choroid plexus net CSF production may be being progressively down regulated, secondary to the dysregulation of its protein synthesis [53]. It is suggested that altered gene expression within the choroid plexus produces the down regulation of CSF production in AD [54]. However, as the choroid produces 70% of the CSF [47], down regulation alone cannot account for the 85% reduction in CSF_fr_ noted in severe AD or the lack of an expected vast increase in CSF_fr_ from a leaky BBB. In MCI, the variable hyper-perfusion of brain regions with an intact BBB (no increased fluid production if the BBB is intact) may be masking the effect of hypo-perfusion in areas with a deficient BBB (hypothesised to reduce fluid production) in this global CBF study. However, the reduction in ICP in MCI suggests that the hypo-perfused areas may be dominating regarding a reduction in the CSF_fr_.

### 4.3. Why Is the Blood–Brain Barrier Disrupted in AD?

The vascular hypothesis of AD was first suggested by de la Torre and Mussivand in 1993 and was a significant departure from the previous amyloid first paradigm [55]. One of the current authors, in 2004, extended the vascular hypothesis to propose that normal aging and senile dementia may be manifestations of a breakdown in arterial pulsation dampening, with either too large an arterial pulsation to be dampened, too small a compliance in the outflow pathways to allow dampening, or a combination of both [56]. It was suggested that a form of pulse-wave encephalopathy could ensue if this dampening failed [56]. This hypothesis was tested in a pilot study of 12 patients with mild-to-moderate AD compared to 12 age-matched controls. The mean arterial pressure was the same as the controls, but the pulse pressure in AD was 9% higher. Using 2D phase contrast MR flow quantification, the arterial pulse volume within the arterial inflow flow was found to be 13.5% less than controls. Dividing the arterial pulse volume by the arterial pulse pressure suggested that the compliance of the arterial tree in AD was reduced by 20% (*p* = 0.05) [12]. Thus, in AD, there is the stiffening of the arterial tree, which may increase the pulse pressure within the capillary bed due to a reduction in the dampening of this pressure from the reduced arterial compliance [12]. The arterial inflow pulse pressure increases with aging because the large elastic and muscular arteries become stiffer and the pulse pressure within the vascular system increases [57]. The cerebral vessels also become stiffer with age [57]. The increased pulse pressure is correlated with cognitive loss and neurodegeneration [57]. A mouse model of carotid stiffness showed that blood–brain barrier disruption occurred as a direct consequence of an increased pulse pressure [58]. This represents the first part of the two-hit hypothesis in AD. According to the two-hit hypothesis, damage to the brain’s microcirculation occurs from aging and vascular risk factors such as hypertension, cerebrovascular disease, diabetes, and hyperlipidaemia, which lead to BBB dysfunction [59]. This leads to the second hit, i.e., increased amyloid β accumulation and impaired clearance [59].

### 4.4. The Cause of the Second Hit

We have suggested that the initiating event in AD is the breakdown of the BBB, secondary to an elevated pulse pressure. The BBB disruption would be expected to increase the risk of toxic chemicals passing into the brain as well as the passage of bacteria and viruses. We suggest that the brain partly mitigates this effect by making itself ischemic to reduce the leakage of these toxins. Unfortunately, this strategy is not without consequences. The prolonged ischemia will eventually lead to the loss of synapses and whole neurones due to the accumulating ischemic damage.

The other problem is the accumulation of amyloid-β precursor protein components within the brain. Low CSF concentrations of amyloid beta (Aβ) products in patients with AD correlates with a high brain deposition, suggesting decreased clearance of these toxins [60]. In early AD, before patients are symptomatic, CSF Aβ is reduced, but there is increased amyloid PET tracer retention [61]. After a variable lag period, neuronal dysfunction and neurodegeneration develop [61]. Weller noted that Aβ appears to accumulate within the perivascular interstitial fluid drainage pathways of the brain [62]. The modern understanding of the interstitial drainage pathways comes from the glymphatic hypothesis. It is thought that CSF passes into the arterial perivascular spaces to percolate through the brain parenchyma and exit via the venous perivascular spaces into the subarachnoid space and also the lymphatics within the dura [63]. Glymphatic fluid flow can be measured using an MRI technique called DTI-ALPS (diffusion tensor imaging along the perivascular space). Using this technique, a reduction in glymphatic function was noted in the preclinical and prodromal stages of AD [64]. A lower ALPS also predicts amyloid deposition, neurodegeneration, and clinical progression [64]. The entrance of the CSF into the periarterial space is thought to require a volume change within the artery, i.e., pulsatile blood flow. As we have noted, the volume change in the artery in AD is reduced due to a lower arterial compliance [12]. Unfortunately, this will be exacerbated by the progressive arterial contraction we hypothesise to occur in AD with disease progression. The contraction of the smooth muscle cells in the arteries increases the wall tension and reduces the vessel compliance regardless of the pressure or diameter of the vessel [65]. Thus, arterial contraction, in an effort to combat the BBB leakage, will decrease the arterial volume pulsation and, therefore, the glymphatic flow, leading to the accumulation of these toxic proteins within the brain.

### 4.5. Clinical Utility

The modelling we have performed suggests that a breakdown of the BBB is the first finding in AD, and the other manifestations occur secondary to this. Thus, we would suggest that strategies aimed at reducing arterial pulse pressure and improving arterial compliance may be of benefit in this disease. The literature, as described, indicates that there is prognostic information within the degree of the blood–brain barrier disruption, the reduction in the intracranial pressure, and the cerebral blood flow. This is because they correlate strongly with cognitive impairment in Alzheimer’s disease. Unfortunately, BBB disruption is difficult to measure routinely, and the ICP measurement requires an invasive lumbar puncture and is prone to variability. Given that cerebral blood flow correlates strongly with the stage of the disease, it may be directly altering the ICP via a moderation of the effects of the BBB. Then, the measurement of the total arterial inflow (which is relatively easy to perform at routine MRI using a phase contrast flow quantification technique) could provide a surrogate marker of disease progression and therapeutic effect in clinical trials of disease-modifying drugs without the need to measure the other two variables.

### 4.6. Limitations

There are many assumptions inherent in lumped parameter modelling. The model averages all of the brain parenchyma together and, therefore, individual segments are not directly modelled. Poiseuille’s equation requires flow through a thin, rigid, and circular tube of a Newtonian fluid without turbulence. To the degree that these assumptions hold, the findings would be accurate. However, despite its limitations, this equation is commonly used in modelling vasculature in the literature.

Some of the data we required for this model are not available from human studies. In their absence, animal studies were utilised. This is exemplified by the data linking the dilatation of the capillaries to the changes in TMP, which was taken from rodent studies and normal venous TMP, which was obtained from primate studies. We have no way of knowing if the animal data closely approximate human findings, so this is a limitation. Despite this, the model seems to correctly predict the CBV in both severe AD and following a tap test.

## 5. Conclusions

The earliest changes in the patients destined to develop AD are vascular stiffening and increased pulse pressure with the disruption of the BBB. We hypothesised that the brain may partly react to this by constricting its arteries to reduce the leakage of interstitial fluid and protein into the parenchyma. This may work for some time and mitigate the risk of BBB disruption. However, eventually, ischemic damage and a reduction in glymphatic flow will result. The later increases amyloid accumulation, leading to further damage.

## Data Availability

All data are contained within the article.
